# 2-Chloro-*N*-[1-(4-chloro­phen­yl)-3-cyano-1*H*-pyrazol-5-yl]acetamide

**DOI:** 10.1107/S1600536812043966

**Published:** 2012-10-31

**Authors:** Hai-ping Mu, Yang Yang, Qiang-hua Jiang, Xiao-dong Fu, Rong Wan

**Affiliations:** a Department of Applied Chemistry, College of Science, Nanjing University of Technology, No. 5 Xinmofan Road, Nanjing, Nanjing 210009, People’s Republic of China

## Abstract

The title compound, C_12_H_8_Cl_2_N_4_O, was synthesized by the reaction of 5-amino-1-(4-chloro­phen­yl)-1*H*-pyrazole-3-carbonitrile and 2-chloro­acetyl chloride. The dihedral angle between the pyrazole and benzene rings is 30.7 (3)°. In the crystal structure, strong N—H⋯O hydrogen bonds link the mol­ecules into chains along [001]. C—H⋯N hydrogen bonds are also present.

## Related literature

The title compound is important in the synthesis of derivatives of the insecticide Fipronil {systematic name: (*RS*)-5-amino-1-[2,6-dichloro-4-(trifluoro­meth­yl)phen­yl]-4-(trifluoro­methyl­sulfin­yl)-1*H*-pyrazole-3-carbonitrile}. For the biological activity of *N*-pyrazole derivatives, see: Zhao *et al.* (2010 ▶); Liu *et al.* (2010 ▶). For bond-length data, see: Allen *et al.* (1987 ▶). For the structure of 2-chloro-*N*-(3-cyano-1-(2,6-dichloro-4-(tri­fluoro­meth­yl)phen­yl)-1*H*-pyrazol-5-yl)acetamide, see: Zhang *et al.* (2012 ▶).
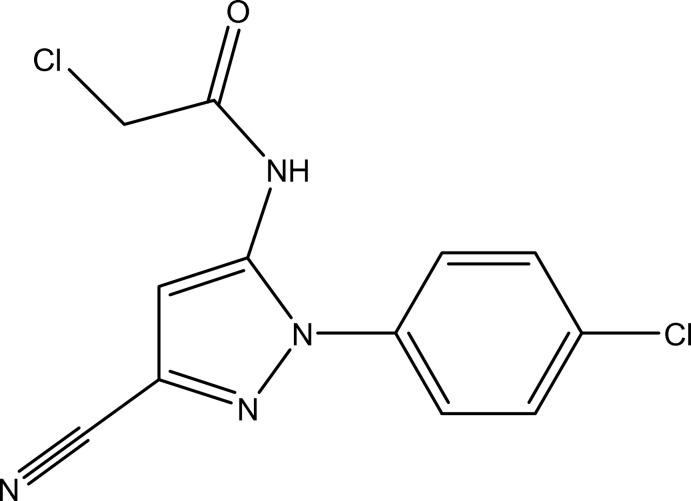


## Experimental

### 

#### Crystal data

C_12_H_8_Cl_2_N_4_O
*M* *_r_* = 295.12
Orthorhombic, 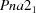

*a* = 18.493 (4) Å
*b* = 13.815 (3) Å
*c* = 5.060 (1) Å
*V* = 1292.7 (4) Å^3^
*Z* = 4
Mo *K*α radiation
μ = 0.50 mm^−1^
*T* = 293 K
0.30 × 0.20 × 0.10 mm

#### Data collection

Enraf–Nonius CAD-4 diffractometer
Absorption correction: ψ scan (North *et al.*, 1968 ▶) *T*_min_ = 0.865, *T*_max_ = 0.952
2646 measured reflections
2606 independent reflections
2255 reflections with *I* > 2σ(*I*)
*R*_int_ = 0.024
3 standard reflections every 200 reflections intensity decay: 1%

#### Refinement

*R*[*F* ^2^ > 2σ(*F* ^2^)] = 0.037
*wR*(*F* ^2^) = 0.099
*S* = 1.01
2606 reflections
173 parameters
1 restraint
H-atom parameters constrained
Δρ_max_ = 0.16 e Å^−3^
Δρ_min_ = −0.24 e Å^−3^
Absolute structure: Flack (1983 ▶), 1271 Friedel pairs
Flack parameter: 0.09 (9)

### 

Data collection: *CAD-4 EXPRESS* (Enraf–Nonius, 1989 ▶); cell refinement: *CAD-4 EXPRESS*; data reduction: *XCAD4* (Harms & Wocadlo, 1995 ▶); program(s) used to solve structure: *SHELXS97* (Sheldrick, 2008 ▶); program(s) used to refine structure: *SHELXL97* (Sheldrick, 2008 ▶); molecular graphics: *SHELXTL* (Sheldrick, 2008 ▶); software used to prepare material for publication: *SHELXS97*.

## Supplementary Material

Crystal structure: contains datablock(s) global, I. DOI: 10.1107/S1600536812043966/rn2106sup1.cif

Structure factors: contains datablock(s) I. DOI: 10.1107/S1600536812043966/rn2106Isup2.hkl

Supplementary material file. DOI: 10.1107/S1600536812043966/rn2106Isup3.cml

Additional supplementary materials:  crystallographic information; 3D view; checkCIF report

## Figures and Tables

**Table 1:** Hydrogen-bond geometry (Å, °)

*D*—H⋯*A*	*D*—H	H⋯*A*	*D*⋯*A*	*D*—H⋯*A*
N3—H3*A*⋯O^i^	0.86	2.16	2.858 (3)	137
C12—H12*C*⋯N2^ii^	0.97	2.52	3.445 (3)	160

Symmetry codes: (i) 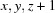; (ii) 
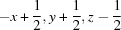
.

**Fig. 1.**
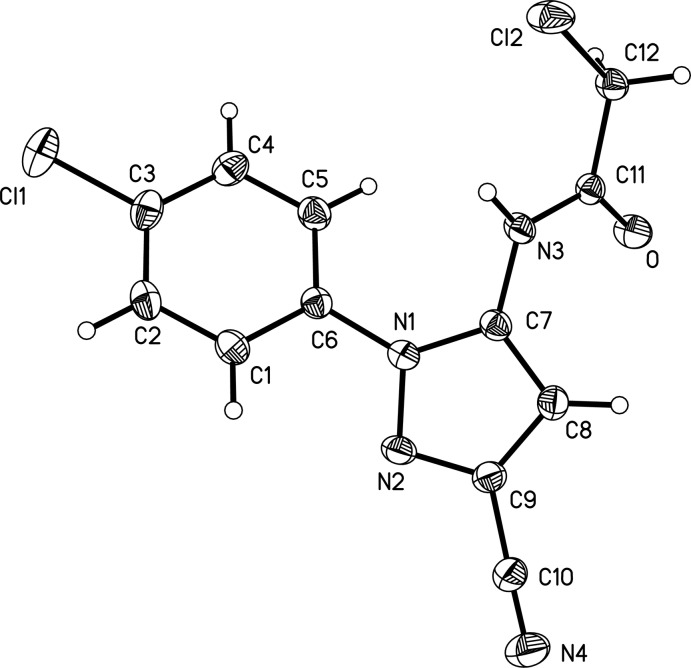
A view of the molecular structure of (I). Displacement ellipsoids are drawn at the 50% probability level.

**Fig. 2.**
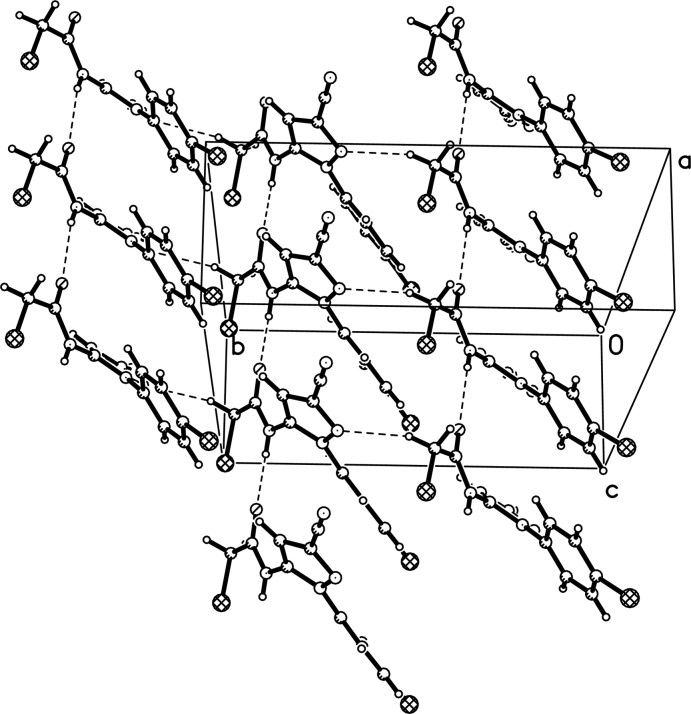
Partial packing view showing the hydrogen-bonded network. Dashed lines indicate intermolecular N—H···O and C—H···N hydrogen bonds.

## supplementary crystallographic information

### Comment

*N*-Pyrazole derivatives are of great interest because of their diverse biological activities such as insecticidal (Zhao *et al.*, 2010) and antifungal activities (Liu *et al.*, 2010). The title compound is an important intermediate in the synthesis of N-aromatic pyrazole derivatives. The molecular structure of (I) is shown in Fig.1. In this structure, bond length and angles are within the normal range (Allen *et al.*, 1987) and the mean deviation from the plane(N1/N2/C9/C8/C7) is 0.0045 Å. The dihedral angle between the pyrazole and phenyl ring in compound (I) is 30.7 (3)°, which is smaller than the angle in the structure of 2-chloro-N- (3-cyano-1-(2,6-dichloro-4-(trifluoromethyl)phenyl)-1*H*-pyrazol-5-yl) acetamide (Zhang *et al.*, 2012), which is 71.5 (3)°. While bond lengths of the two compounds are similar, the difference in the dihedral angle probably results from greater steric hindrance in the (trifluoromethyl)phenyl derivative. In the crystal structure, strong N—H···O hydrogen bonds link the molecules into infinite one-dimensional chains along the [001] direction. Intermolecular C—H···N and N—H···O hydrogen bonds (Table 1) may help to establish the molecular conformation of (I). (Fig. 2)

### Experimental

To a stirred solution of 5-amino-1-(4-chlorophenyl)-1*H*-pyrazole-3-carbonitrile (5 mmol) in THF (20 ml) was added 2-chloroacetyl chloride (5 mmol) dropwise at 0–5°C. During the addition, the solution is cooled in an ice-salt bath. After the cooling bath had been removed, the reaction mixture was allowed to stand for 2 h at room temperature. The crude product (I) precipitated and was filtered. Pure compound (I) was obtained by crystallization from ethanol. Crystals of (I) suitable for X-ray diffraction were obtained by slow evaporation of an acetone solution.

### Refinement

All H atoms bonded to the C atoms were placed geometrically at the distances of 0.93–0.97 Å and included in the refinement in riding motion approximation with *U*_iso_(H) = 1.2 or 1.5*U*_eq_ of the carrier atom.

### Crystal data

**Table:**

C_12_H_8_Cl_2_N_4_O	*D*_x_ = 1.516 Mg m^−^^3^
*M**_r_* = 295.12	Mo *K*α radiation, λ = 0.71073 Å
Orthorhombic, *P**n**a*2_1_	Cell parameters from 25 reflections
*a* = 18.493 (4) Å	θ = 9–13°
*b* = 13.815 (3) Å	µ = 0.50 mm^−^^1^
*c* = 5.060 (1) Å	*T* = 293 K
*V* = 1292.7 (4) Å^3^	Block, colorless
*Z* = 4	0.30 × 0.20 × 0.10 mm
*F*(000) = 600	

### Data collection

**Table:**

Enraf–Nonius CAD-4 diffractometer	2255 reflections with *I* > 2σ(*I*)
Radiation source: fine-focus sealed tube	*R*_int_ = 0.024
Graphite monochromator	θ_max_ = 25.4°, θ_min_ = 2.2°
ω/2θ scans	*h* = −22→22
Absorption correction: ψ scan (North *et al.*, 1968)	*k* = 0→16
*T*_min_ = 0.865, *T*_max_ = 0.952	*l* = −6→0
2646 measured reflections	3 standard reflections every 200 reflections
2606 independent reflections	intensity decay: 1%

### Refinement

**Table:**

Refinement on *F*^2^	Hydrogen site location: inferred from neighbouring sites
Least-squares matrix: full	H-atom parameters constrained
*R*[*F*^2^ > 2σ(*F*^2^)] = 0.037	*w* = 1/[σ^2^(*F*_o_^2^) + (0.064*P*)^2^] where *P* = (*F*_o_^2^ + 2*F*_c_^2^)/3
*wR*(*F*^2^) = 0.099	(Δ/σ)_max_ < 0.001
*S* = 1.01	Δρ_max_ = 0.16 e Å^−^^3^
2606 reflections	Δρ_min_ = −0.24 e Å^−^^3^
173 parameters	Extinction correction: *SHELXL97* (Sheldrick, 2008), Fc^*^=kFc[1+0.001xFc^2^λ^3^/sin(2θ)]^-1/4^
1 restraint	Extinction coefficient: 0.023 (2)
Primary atom site location: structure-invariant direct methods	Absolute structure: Flack (1983), 1271 Friedel pairs
Secondary atom site location: difference Fourier map	Flack parameter: 0.09 (9)

### Special details

**Table:**

Geometry. All e.s.d.'s (except the e.s.d. in the dihedral angle between two l.s. planes) are estimated using the full covariance matrix. The cell e.s.d.'s are taken into account individually in the estimation of e.s.d.'s in distances, angles and torsion angles; correlations between e.s.d.'s in cell parameters are only used when they are defined by crystal symmetry. An approximate (isotropic) treatment of cell e.s.d.'s is used for estimating e.s.d.'s involving l.s. planes.
Refinement. Refinement of *F*^2^ against ALL reflections. The weighted *R*-factor *wR* and goodness of fit *S* are based on *F*^2^, conventional *R*-factors *R* are based on *F*, with *F* set to zero for negative *F*^2^. The threshold expression of *F*^2^ > σ(*F*^2^) is used only for calculating *R*-factors(gt) *etc*. and is not relevant to the choice of reflections for refinement. *R*-factors based on *F*^2^ are statistically about twice as large as those based on *F*, and *R*- factors based on ALL data will be even larger.

### Fractional atomic coordinates and isotropic or equivalent isotropic displacement parameters (Å^2^)

**Table:**

	*x*	*y*	*z*	*U*_iso_*/*U*_eq_	
O	0.20761 (11)	0.90238 (15)	−0.4786 (4)	0.0473 (5)	
Cl1	0.07270 (4)	0.51152 (5)	0.76028 (19)	0.0634 (3)	
N1	0.27787 (9)	0.73978 (13)	0.1128 (5)	0.0330 (5)	
C1	0.25223 (13)	0.63629 (18)	0.4875 (6)	0.0385 (6)	
H1A	0.3011	0.6381	0.5309	0.046*	
Cl2	0.07546 (4)	0.99063 (6)	0.07496 (17)	0.0548 (2)	
N2	0.34595 (10)	0.70361 (14)	0.0940 (6)	0.0397 (5)	
C2	0.20505 (14)	0.58124 (18)	0.6366 (6)	0.0428 (7)	
H2B	0.2220	0.5449	0.7785	0.051*	
N3	0.20572 (11)	0.87436 (15)	−0.0382 (5)	0.0339 (5)	
H3A	0.1824	0.8829	0.1070	0.041*	
C3	0.13257 (15)	0.58071 (18)	0.5729 (6)	0.0432 (6)	
C4	0.10698 (14)	0.63335 (19)	0.3602 (7)	0.0442 (7)	
H4A	0.0579	0.6325	0.3198	0.053*	
N4	0.51184 (12)	0.73394 (18)	−0.1943 (8)	0.0707 (9)	
C5	0.15393 (13)	0.68719 (18)	0.2079 (6)	0.0407 (6)	
H5A	0.1370	0.7220	0.0632	0.049*	
C6	0.22697 (12)	0.68864 (16)	0.2739 (6)	0.0330 (6)	
C7	0.27029 (12)	0.82144 (17)	−0.0352 (5)	0.0329 (6)	
C8	0.33442 (12)	0.83866 (18)	−0.1594 (6)	0.0388 (6)	
H8A	0.3457	0.8887	−0.2752	0.047*	
C9	0.37887 (13)	0.76412 (17)	−0.0731 (6)	0.0390 (6)	
C10	0.45341 (15)	0.74665 (18)	−0.1393 (8)	0.0492 (8)	
C11	0.17925 (12)	0.91243 (17)	−0.2649 (5)	0.0314 (5)	
C12	0.10972 (13)	0.96827 (18)	−0.2494 (6)	0.0357 (6)	
H12B	0.0732	0.9332	−0.3483	0.043*	
H12C	0.1168	1.0301	−0.3365	0.043*	

### Atomic displacement parameters (Å^2^)

**Table:**

	*U*^11^	*U*^22^	*U*^33^	*U*^12^	*U*^13^	*U*^23^
O	0.0494 (11)	0.0672 (14)	0.0253 (11)	0.0153 (10)	0.0035 (9)	0.0035 (9)
Cl1	0.0730 (5)	0.0656 (5)	0.0516 (5)	−0.0249 (4)	0.0147 (5)	0.0028 (5)
N1	0.0321 (10)	0.0332 (10)	0.0337 (13)	0.0041 (8)	−0.0021 (10)	−0.0001 (11)
C1	0.0422 (14)	0.0375 (14)	0.0358 (16)	0.0021 (11)	−0.0055 (13)	−0.0011 (12)
Cl2	0.0469 (4)	0.0817 (5)	0.0359 (4)	0.0234 (3)	0.0066 (4)	−0.0023 (4)
N2	0.0315 (10)	0.0376 (10)	0.0499 (15)	0.0067 (8)	−0.0030 (11)	0.0018 (13)
C2	0.0568 (16)	0.0392 (13)	0.0324 (16)	0.0039 (12)	−0.0011 (13)	0.0053 (13)
N3	0.0391 (11)	0.0391 (12)	0.0235 (11)	0.0119 (9)	0.0029 (10)	0.0032 (10)
C3	0.0561 (16)	0.0395 (13)	0.0341 (15)	−0.0064 (11)	0.0088 (15)	−0.0043 (14)
C4	0.0385 (14)	0.0512 (16)	0.0430 (17)	−0.0066 (12)	0.0014 (14)	−0.0034 (15)
N4	0.0401 (14)	0.0684 (16)	0.104 (3)	0.0034 (11)	0.0086 (17)	0.008 (2)
C5	0.0430 (14)	0.0443 (14)	0.0348 (16)	0.0042 (11)	−0.0031 (12)	0.0025 (13)
C6	0.0360 (12)	0.0303 (11)	0.0328 (14)	0.0022 (9)	0.0007 (11)	−0.0015 (12)
C7	0.0346 (12)	0.0344 (13)	0.0298 (13)	0.0039 (10)	−0.0011 (12)	−0.0003 (12)
C8	0.0409 (13)	0.0370 (13)	0.0384 (16)	0.0004 (11)	0.0043 (13)	0.0043 (13)
C9	0.0324 (12)	0.0382 (13)	0.0465 (17)	−0.0010 (11)	0.0011 (13)	−0.0002 (13)
C10	0.0397 (14)	0.0411 (15)	0.067 (2)	−0.0002 (11)	0.0052 (15)	0.0078 (16)
C11	0.0344 (12)	0.0338 (12)	0.0259 (13)	0.0009 (9)	−0.0002 (12)	0.0016 (12)
C12	0.0366 (12)	0.0425 (13)	0.0279 (13)	0.0059 (10)	−0.0019 (12)	0.0030 (13)

### Geometric parameters (Å, º)

**Table:**

O—C11	1.210 (3)	N3—H3A	0.8600
Cl1—C3	1.743 (3)	C3—C4	1.382 (4)
N1—N2	1.358 (2)	C4—C5	1.379 (4)
N1—C7	1.361 (3)	C4—H4A	0.9300
N1—C6	1.431 (3)	N4—C10	1.129 (3)
C1—C6	1.382 (4)	C5—C6	1.391 (3)
C1—C2	1.382 (4)	C5—H5A	0.9300
C1—H1A	0.9300	C7—C8	1.363 (3)
Cl2—C12	1.786 (3)	C8—C9	1.388 (3)
N2—C9	1.336 (4)	C8—H8A	0.9300
C2—C3	1.379 (4)	C9—C10	1.439 (4)
C2—H2B	0.9300	C11—C12	1.502 (3)
N3—C11	1.354 (3)	C12—H12B	0.9700
N3—C7	1.400 (3)	C12—H12C	0.9700
			
N2—N1—C7	111.21 (19)	C1—C6—C5	120.6 (2)
N2—N1—C6	117.91 (19)	C1—C6—N1	118.8 (2)
C7—N1—C6	130.88 (18)	C5—C6—N1	120.6 (2)
C6—C1—C2	120.1 (2)	N1—C7—C8	108.0 (2)
C6—C1—H1A	119.9	N1—C7—N3	121.8 (2)
C2—C1—H1A	119.9	C8—C7—N3	130.2 (2)
C9—N2—N1	103.70 (19)	C7—C8—C9	103.9 (2)
C3—C2—C1	119.3 (3)	C7—C8—H8A	128.0
C3—C2—H2B	120.4	C9—C8—H8A	128.0
C1—C2—H2B	120.4	N2—C9—C8	113.2 (2)
C11—N3—C7	121.4 (2)	N2—C9—C10	118.6 (2)
C11—N3—H3A	119.3	C8—C9—C10	128.2 (3)
C7—N3—H3A	119.3	N4—C10—C9	179.0 (4)
C2—C3—C4	120.8 (3)	O—C11—N3	123.8 (2)
C2—C3—Cl1	119.6 (2)	O—C11—C12	118.5 (2)
C4—C3—Cl1	119.6 (2)	N3—C11—C12	117.7 (2)
C5—C4—C3	120.2 (2)	C11—C12—Cl2	116.15 (19)
C5—C4—H4A	119.9	C11—C12—H12B	108.2
C3—C4—H4A	119.9	Cl2—C12—H12B	108.2
C4—C5—C6	119.0 (3)	C11—C12—H12C	108.2
C4—C5—H5A	120.5	Cl2—C12—H12C	108.2
C6—C5—H5A	120.5	H12B—C12—H12C	107.4
			
C7—N1—N2—C9	−1.3 (3)	C6—N1—C7—C8	−177.8 (3)
C6—N1—N2—C9	178.0 (2)	N2—N1—C7—N3	−177.1 (2)
C6—C1—C2—C3	−1.2 (4)	C6—N1—C7—N3	3.8 (4)
C1—C2—C3—C4	0.9 (4)	C11—N3—C7—N1	−139.4 (3)
C1—C2—C3—Cl1	−179.9 (2)	C11—N3—C7—C8	42.6 (4)
C2—C3—C4—C5	0.2 (4)	N1—C7—C8—C9	−0.7 (3)
Cl1—C3—C4—C5	−179.0 (2)	N3—C7—C8—C9	177.5 (3)
C3—C4—C5—C6	−0.9 (4)	N1—N2—C9—C8	0.8 (3)
C2—C1—C6—C5	0.5 (4)	N1—N2—C9—C10	−179.9 (3)
C2—C1—C6—N1	−175.9 (2)	C7—C8—C9—N2	−0.1 (3)
C4—C5—C6—C1	0.5 (4)	C7—C8—C9—C10	−179.3 (3)
C4—C5—C6—N1	176.9 (2)	N2—C9—C10—N4	169 (22)
N2—N1—C6—C1	29.2 (3)	C8—C9—C10—N4	−12 (22)
C7—N1—C6—C1	−151.8 (3)	C7—N3—C11—O	1.6 (4)
N2—N1—C6—C5	−147.2 (2)	C7—N3—C11—C12	−179.9 (2)
C7—N1—C6—C5	31.8 (4)	O—C11—C12—Cl2	−174.0 (2)
N2—N1—C7—C8	1.3 (3)	N3—C11—C12—Cl2	7.4 (3)

### Hydrogen-bond geometry (Å, º)

**Table:**

*D*—H···*A*	*D*—H	H···*A*	*D*···*A*	*D*—H···*A*
N3—H3*A*···O^i^	0.86	2.16	2.858 (3)	137
C12—H12*C*···N2^ii^	0.97	2.52	3.445 (3)	160

Symmetry codes: (i) *x*, *y*, *z*+1; (ii) −*x*+1/2, *y*+1/2, *z*−1/2.

## References

[bb1] Allen, F. H., Kennard, O., Watson, D. G., Brammer, L., Orpen, A. G. & Taylor, R. (1987). *J. Chem. Soc. Perkin Trans. 2*, pp. S1–19.

[bb2] Enraf–Nonius (1989). *CAD-4 Software* Enraf–Nonius, Delft, The Netherlands.

[bb3] Flack, H. D. (1983). *Acta Cryst.* A**39**, 876–881.

[bb4] Harms, K. & Wocadlo, S. (1995). *XCAD4* University of Marburg, Germany.

[bb5] Liu, Y. Y., Shi, H., Li, Y. F. & Zhu, H. J. (2010). *J. Heterocycl. Chem* **47**, 897–902.

[bb6] North, A. C. T., Phillips, D. C. & Mathews, F. S. (1968). *Acta Cryst.* A**24**, 351–359.

[bb7] Sheldrick, G. M. (2008). *Acta Cryst.* A**64**, 112–122. 10.1107/S0108767307043930 18156677

[bb8] Zhang, J., He, Q., Jiang, Q., Mu, H. & Wan, R. (2012). *Acta Cryst.* E**68**, o104. 10.1107/S1600536811052743 PMC3254452 22259391

[bb9] Zhao, Q. Q., Li, Y. Q., Xiong, L. X. & Wang, Q. M. (2010). *J. Agric. Food Chem.* **58**, 4992–4998. 10.1021/jf1001793 20349960

